# Agriculture and health: synergizing for a sustainable future

**DOI:** 10.3389/fpubh.2025.1558198

**Published:** 2025-04-02

**Authors:** Enrique Teran, Morgan Chetty, Sara Touirsi

**Affiliations:** ^1^Colegio de Ciencias de la Salud, Universidad San Francisco de Quito, Quito, Ecuador; ^2^African Global Health Ambassador, Casablanca, Morocco; ^3^KZNDHC and IPA Foundation of SA, Member of International Association of Quality and Safety (IAQS), Cape Town, South Africa

**Keywords:** agriculture, health, sustainable development, south cooperation, diseases prevention

## Introduction

Agriculture and health are two of the most critical issues in sustainability. Over time, it has been observed that advances in agriculture have brought forth significant changes in people's nutritional status and health conditions. Traditional agricultural practices offer benefits such as low input costs, biodiversity conservation, and reliance on Indigenous knowledge, enhancing ecological resilience (1). However, they also present significant challenges, like continuous monocropping and intensive tillage (2), overuse of pesticides (3), a limited number of staple crops (4), and development of crop varieties tolerant to adverse conditions (5), underscore the need for sustainable and innovative approaches.

Improved agricultural practices have led to increased food production, which has, in turn, enhanced the availability and accessibility of nutritious food. This positive shift has contributed to reducing hunger and malnutrition in many parts of the world. However, contemporary issues like climate change, population increase, and the emergence of non-communicable diseases (NCDs) require a comprehensive re-evaluation of the potential of agriculture to improve public health (6).

Climate change poses a serious threat to agricultural productivity by altering weather patterns, reducing crop yields, and increasing the prevalence of pests and diseases. These changes can lead to food insecurity, which directly impacts health by increasing the risk of malnutrition and related health conditions. Moreover, the global population is expected to reach 9.7 billion by 2050, which will significantly increase the demand for food. Meeting this demand sustainably will be a major challenge, especially in regions already struggling with food security (6).

Additionally, the rise of NCDs such as diabetes, heart disease, and obesity are partly attributable to poor dietary habits. Addressing these health challenges requires a multifaceted approach that includes promoting healthy eating through sustainable agricultural practices. For instance, diversifying crop production to include more fruits, vegetables, and legumes can enhance dietary quality and help prevent NCDs (7).

The purpose of this manuscript is to explore how agricultural food security can promote health and reduce diseases. This underscores the need for integrated policies that tackle agriculture and health as interconnected issues. By adopting an integrated approach, it is possible to create a synergistic relationship between agriculture and health, leading to a sustainable and healthy future for all.

## Relationship between agriculture and health

Nutrition plays a significant role in the quality of one's life. Substantial progress has been made in halving food insecurity; unfortunately, poor eating habits remain the largest threat to health and longevity across the globe. Cardiovascular diseases, cancers, and type-2 diabetes are some of the diet-related diseases that are prevalent. The cases of obesity due to improper diet add to these complications solely due to the rampant cases of improper meal intake. It was established that diet-related diseases are a leading cause of death, accounting for 11 million deaths per year, which implies the necessity to improve the quality of food production and consumption (7).

Furthermore, inadequate dietary diversity and reliance on energy-dense, nutrient-poor foods exacerbate these health issues. The overconsumption of processed foods high in sugars, fats, and salts is a significant contributor to the rise of NCDs. This dietary shift, often referred to as the “nutrition transition,” is increasingly common in low- and middle-income countries as they undergo economic and social changes (8). Consequently, there is an urgent need to promote dietary patterns that emphasize whole grains, fruits, vegetables, nuts, and legumes to combat the growing burden of NCDs.

It is factual that farm correlates with the nutritional status of the people. Internationally, over 800 million people are undernourished; they lack adequate macronutrient and micronutrient intake (9). This undernutrition is often due to food insecurity, which can result from poor agricultural productivity, economic instability, and climate-related impacts on crop yields. Additionally, the “hidden hunger” caused by micronutrient deficiencies, such as iron, vitamin A, and iodine, affects a large portion of the global population, leading to serious health consequences like impaired cognitive development, weakened immunity, and increased maternal and child mortality (10).

On the same note, the present-day global adult population is estimated to be half of it being overweight or obese, resulting in a global outbreak of NCDs including diabetes and hypertension, among others. Stand-alone synergistic diseases, undernutrition, and overnutrition are the most threatening health issues in the present world. These challenges must be solved through a complex intervention that entails increasing agricultural productivity and access to healthy foods (11).

Increased agricultural productivity, however, must be accompanied by strategies to ensure that the food produced is nutritious and accessible. Efforts such as biofortification, which involves producing crops to increase their nutritional value, and improving post-harvest processing to preserve nutrient content, are essential. For example, biofortified crops like vitamin A-rich sweet potatoes and iron-fortified beans have shown promise in addressing micronutrient deficiencies (12).

Agriculture helps conserve soil health, reduce water pollution, minimize greenhouse gas emissions, and preserve biodiversity. Then, sustainable agriculture focuses on producing long-term crops and livestock while having minimal effects on the environment, conserving water, and reducing the use of fertilizers and pesticides (13).

Moreover, sustainable agricultural practices can play a pivotal role in enhancing food security and nutrition. Techniques such as agroecology, which integrates ecological principles into agricultural production, can improve soil health, increase biodiversity, and reduce dependency on chemical inputs, thereby promoting a more resilient food system (14).

The impact of sustainable agriculture extends beyond the environment. While the market value of the Global Food system is estimated at USD 10 trillion, the hidden often externalized costs are USD 12 trillion. Of these massive costs, more than half, i.e., USD 6.6 trillion are related to health (15).

**Figure 1:**
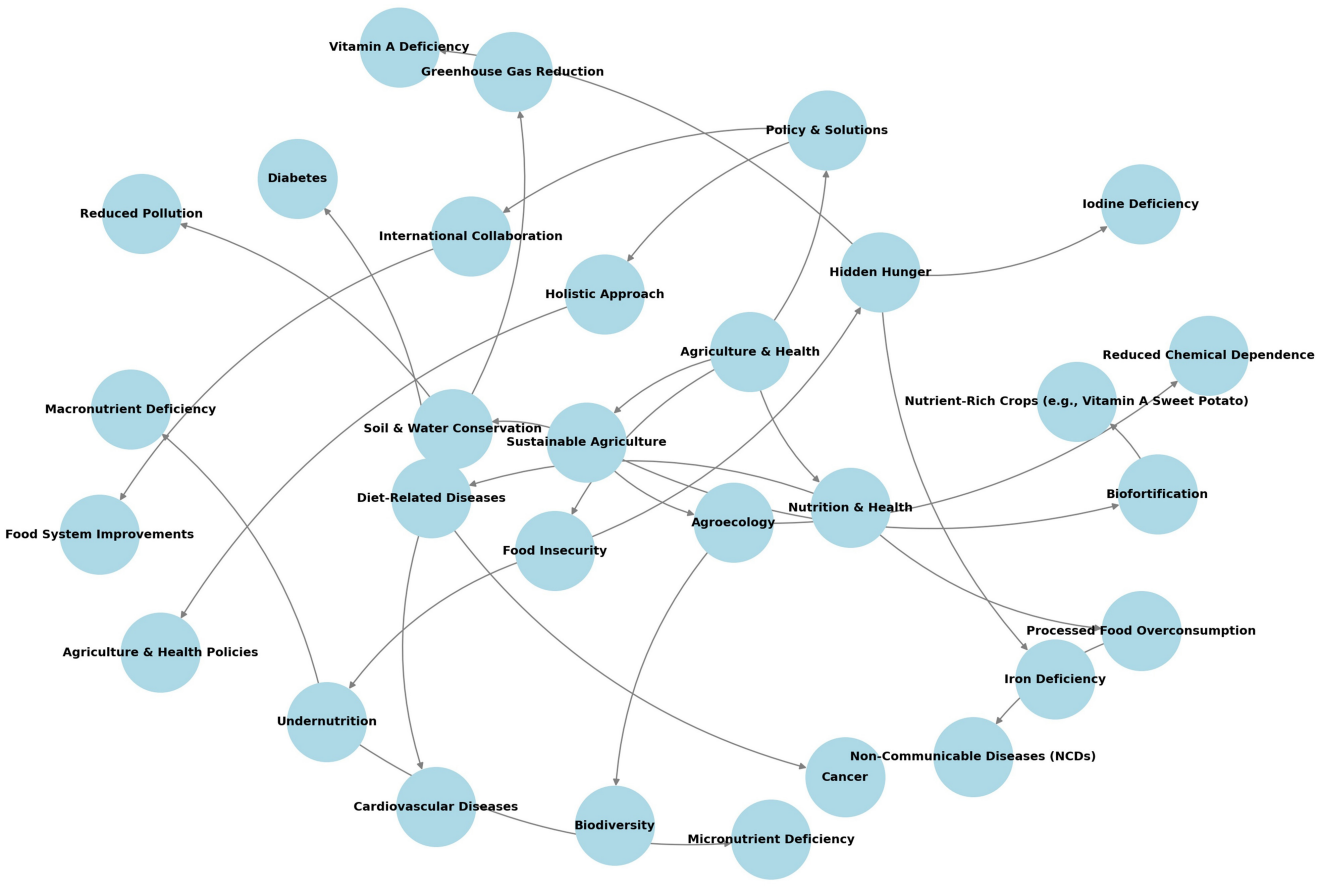
Schematic interactions between agriculture and health.

To address these intertwined issues effectively, it is crucial to adopt a comprehensive approach that integrates agricultural and health policies. Governments and international organizations need to collaborate on creating and implementing policies that promote sustainable agriculture, improve food systems, and ensure that all individuals have access to nutritious, safe, and affordable food. This approach will help mitigate the health impacts of both undernutrition and overnutrition, leading to a healthier global population (Figure 1).

## Agricultural innovations for disease prevention

Modern methods applied in farming systems are critical in combating diseases. The use of technologies such as precision farming and genetically modified organisms (GMOs) has been found to lower the impact of diseases associated with poor diets. For example, decision support systems in precision agriculture define the right amounts of fertilizers needed and where to apply them, thus enhancing the ratios of resource use to food production while at the same time reducing environmental devastation (16).

Precision farming leverages data analytics, remote sensing, and GPS technology to optimize field-level management regarding crop farming. This method helps in the efficient use of inputs such as water, fertilizers, and pesticides, thereby minimizing waste and environmental harm while maximizing crop yields. By precisely targeting the needs of plants, precision farming can significantly enhance the nutritional quality of crops, thus contributing to better health outcomes. Studies have shown that precision farming can increase crop yields by 20–30% and reduce input costs by 10–20% (17).

In cases where GMOs are developed and applied properly, they contribute to increasing the production and quality of food and solve the problem of deficiencies. GMOs can be engineered to be more resistant to pests, diseases, and harsh environmental conditions, which ensures a more stable food supply. Moreover, biofortified GMOs can address specific nutritional deficiencies; for instance, Golden Rice, which is fortified with vitamin A, has been developed to combat vitamin A deficiency, a major cause of blindness and mortality among children in developing countries (18).

Besides, practices such as crop rotation, organic farming, and Integrated Pest Management (IPM) enhance environmental quality and long-run agricultural output. Crop rotation helps in maintaining soil health and reducing pest and disease cycles. Organic farming avoids synthetic chemicals, thereby reducing pollution and conserving biodiversity. IPM combines biological, cultural, and chemical tools to manage pests in an environmentally and economically sustainable way. These sustainable agricultural practices not only protect the environment but also contribute to the production of healthier food, which is essential for preventing diet-related diseases (14).

Furthermore, agroecology, which integrates ecological principles into agricultural practices, has gained attention as a sustainable approach to farming. Agroecological practices such as polycultures, agroforestry, and cover cropping can enhance biodiversity, improve soil health, and increase resilience to climate change. These practices support a more diverse and nutritionally adequate food supply, which is crucial for addressing both undernutrition and overnutrition (19).

In conclusion, modern agricultural innovations play a pivotal role in disease prevention by improving the quality and quantity of food production while minimizing environmental impacts. Through the adoption of precision farming, GMOs, and sustainable agricultural practices, it is possible to enhance food security, improve nutritional outcomes, and promote overall health.

## Conclusion

Returning the focus to agriculture, it is impossible to overlook the fact that agricultural productivity has a direct impact on the health of the population, availability of adequate nutrition, and prevention of diseases. Thus, through promoting effective agricultural practices or including nutrition in health care strategies, societies can resolve the issue of the twin burden of malnutrition and enhance people's quality of life. To fully harness the health impacts of the enhancement made in agriculture, further study and formulation of policies are critical to fashion a healthy population that can greatly support the health services provided by Corporate Social Responsibility as per Carroll's pyramid proposal (20).
